# Effects of a medication adherence app among medically underserved adults with chronic illness: a randomized controlled trial

**DOI:** 10.1007/s10865-023-00446-2

**Published:** 2023-12-21

**Authors:** Christa E. Hartch, Mary S. Dietrich, B. Jeanette Lancaster, Deonni P. Stolldorf, Shelagh A. Mulvaney

**Affiliations:** 1 Vanderbilt University School of Nursing, 461 21st Ave S, Nashville, TN 37240 USA; 2 Present Address: School of Nursing and Health Sciences, Manhattanville College, 2900 Purchase Street, Purchase, NY 10577 USA; 3 Department of Biostatistics, Vanderbilt University Medical Center, 2525 West End Avenue, Suite 1100, Nashville, TN 37203 USA; 4 Sadie Heath Cabiness Professor and Dean Emerita, School of Nursing, University of Virginia, 225 Jeanette Lancaster Way, Charlottesville, VA 22903 USA; 5 Department of Biomedical Informatics, Vanderbilt University Medical Center, 2525 West End Avenue, #1475, Nashville, TN 37203 USA

**Keywords:** Medication adherence, Medication self-efficacy, Mobile phone applications, Medically underserved populations

## Abstract

For individuals living with a chronic illness who require use of long-term medications, adherence is a vital aspect of successful symptom management and outcomes. This study investigated the effect of a smartphone app on adherence, self-efficacy, knowledge, and medication social support in a medically underserved adult population with various chronic illnesses. Participants were randomized to a group who used the app for one month or a control group provided with a printed medication list. Compared to the control group, participants receiving the intervention had significantly greater medication adherence (Cohen’s *d* = -0.52, *p* = .014) and medication self-efficacy (Cohen’s *d* = 0.43, *p* = .035). No significant effects were observed related to knowledge or social support. The findings suggest use of the app could positively impact chronic disease management in a medically underserved population in the United States.

Inadequate adherence to medications in patients who require long-term medication treatment of chronic diseases is a global concern (Brown & Bussell, 2011; Naderi et al., 2012). Inadequate medication adherence results in worsening symptoms and disease severity, complications, and potential mortality, resulting in a less healthy and productive society throughout the world (Chisholm-Burns & Spivey, 2012). Domestically, the prevalence of chronic diseases such as heart disease and diabetes are increasing. It is estimated 60% of adults in the United States (US) have one chronic disease while 40% have two or more (National Center for Chronic Disease Prevention and Health Promotion, 2019). Furthermore, the lifetime disease progression trajectory, where a person progresses from a single chronic disease to comorbidity and requires more complex treatment, adds to the utilization of greater healthcare resources (Zhu et al., 2018).

Interventions to improve medication adherence for patients with chronic illnesses are multifaceted and approaches have been organized into informational, behavioral, and family/social interventions (Kripalani et al., 2007). Informational interventions include cognitive strategies to educate and motivate patients, behavioral interventions may influence behavior through shaping, reminding, and reinforcing desired behaviors, and social interventions typically involve social support strategies (Kripalani et al., 2007). Successful interventions often include more than one approach, or component, which can be resource intensive from a healthcare perspective (Thakkar et al., 2016). For example, a review of medication adherence interventions in chronic care concluded the most effective interventions were complex and included tailored ongoing support from health care providers (HCPs), with intense education, counseling and/or daily treatment support, and some included support from family and/or peers. Even these did not show large improvements in adherence or clinical outcomes over a wide variety of chronic illnesses (Nieuwlaat et al., 2014).

Technological tools, such as mobile phone applications (apps), have shown promise as a tool to help patients adhere to medications (Peng et al., 2020). Most apps focused on chronic illness adherence use behavioral approaches such as tracking and alerts to take a medication and contain information about the medication (Eysenbach et al., 2019). The Medisafe app, a commercially available medication adherence app, incorporates informational, behavioral, and social support components, and is highly rated in both a quality assessment and content analysis of user reviews (Eysenbach et al., 2019; Parra-Calderón et al., 2016). Medisafe was rated as the top mobile app to support medication adherence in a review that evaluated app strengths and weaknesses (Sharma et al., 2022). Although apps have shown promise overall, few studies of medication adherence apps isolate specific features of the app and their effect on medication adherence.

Lower income, medically underserved communities have higher rates of inadequate adherence to oral medications (Bidwal et al., 2017; Bowry et al., 2011; Fernandez-Lazaro et al., 2019; Osborn et al., 2014). The reasons for this are multifaceted and can be influenced by social determinants of health (SDOH) which contribute to health disparities (Office of Disease Prevention and Health Promotion, 2023). SDOH refer to the material and social conditions in which people live (Reutter & Kushner, 2010), and adverse SDOH are associated with lower medication adherence (Wilder et al., 2021). For example, lack of health insurance, food insecurity, and housing instability have been shown to negatively impact medication adherence (Agarwal et al., 2018; Wilder et al., 2021). Other types of barriers to medication adherence in the uninsured with multiple chronic conditions include forgetting and not having enough information about their current medications, both of which may be positively influenced by a medication adherence app (Fernandez-Lazaro et al., 2019).

Mobile apps have been cited as a promising way to reach underserved populations by the potential to increase healthcare access and quality and improve social support (Anderson-Lewis et al., 2018; Peek, 2017). Smartphone ownership among lower-income Americans is increasing and is currently estimated to be 76% (Pew Research Center, 2021). However, medically underserved, low-income populations have been underrepresented in studies utilizing mobile health (mHealth) interventions (Hincapie et al., 2019; Nelson et al., 2016). Including medically underserved individuals in mHealth studies provides opportunities to support this disproportionately affected group, work toward reducing health disparities in access to healthcare, and understand barriers to mHealth uptake (Anderson-Lewis et al., 2018; Peek, 2017).

Additionally, there is a high prevalence of chronic conditions among medically underserved populations (Fernandez-Lazaro et al., 2019), many of whom receive care at Federally Qualified Health Centers (FQHCs). When compared to the overall US population, community health center patients have higher rates of hypertension (HTN) (32% vs. 45%), high cholesterol (36% vs. 42%), asthma (14% vs. 21%) and diabetes (11% vs. 21%) (National Association of Community Health Centers, 2019a). FQHCs are reporting a growth in the treatment of complex chronic conditions, many of which overlap, and patients have been found to have higher rates of chronic conditions at FQHCs than those seen by private practice providers (Corallo et al., 2020; National Association of Community Health Centers, 2019b). A recent randomized controlled trial (RCT) focused on the impact of mobile app on adults diagnosed with comorbid chronic illnesses who were privately insured. Compared to a control group, participants in the app group had greater improvements in objectively measured medication adherence after two, three and twelve months (Li et al., 2021). The prevalence of multiple chronic illnesses and initial limited support highlight the need to study medication adherence app interventions within medically underserved populations with multiple chronic illnesses in the US (Fernandez-Lazaro et al., 2019).

Social cognitive theory posits that self-efficacy beliefs play an important role in the adoption and continuation of health behaviors. Medication self-efficacy is the confidence in one’s ability to take one’s medications and is an important determinant of medication adherence (Risser et al., 2007). Generally, higher medication self-efficacy beliefs are associated with higher medication adherence (Bandura, 2004; Flower et al., 2016; Huang et al., 2018; Lee et al., 2013; Meinema et al., 2015). Surprisingly, few studies have documented whether the use of a medication adherence mobile app increases medication self-efficacy. In a population of women of African descent with lupus in Barbados, where only 8% of patients had private insurance and 2/3 received care at a public clinic, there was a strong association noted between feelings of medication self-efficacy and adherence (Flower et al., 2016). One study conducted in a predominantly White educated population with type 2 diabetes (T2D) measured medication self-efficacy in relation to using an app and showed no effect (*r* = .02) (Kjos et al., 2019). However, an RCT conducted in Vietnam demonstrated the effectiveness of an app on health behaviors and adherence to HIV medications among participants, with HIV adherence self-efficacy increasing significantly after three months of app use (Tran et al., 2023). An RCT conducted in India on adherence to epilepsy medications found that medication self-efficacy for the management of epilepsy also increased significantly after three months of use (Mirpuri et al., 2021). Neither of these studies examined app features separately in relation to self-efficacy or outcomes. Given the importance of self-efficacy in health behavior change, it is important to incorporate this construct in the design and evaluation of mobile apps, and generalizing this construct in medically underserved patients in the US will add to the body of knowledge.

Medication knowledge as a concept can be operationalized in a number of ways, but for this study we assessed this concept using a self-report survey which assessed if the participant can describe how and when to use all of their prescribed medications, medication interactions with other medications and food and beverages, and understanding the possible side effects of medications (Okere et al., 2014). Studies generally show that interventions that attempt to improve medication knowledge in chronic illnesses such as T2D (Muhammad Haskani et al., 2022) and HTN (Tan et al., 2019) do not lead to higher medication adherence. For example, two RCTs in T2D, one which used a structured tool within the electronic health record (EHR) to organize patient/provider interventions (Graumlich et al., 2016) and one which used an interactive tailored iPad decision aid (Hofer et al., 2017), suggest improved medication knowledge alone is not sufficient to improve medication adherence. In a chronic pain sample, Timmerman et al., 2016 used an RCT to test efficacy of an intervention where the intervention group watched a video of a physician discussing the medication name, common frequencies, the type of medication, mode, speed of action and common side effects and received medication-specific written information compared to a standard care group. The authors found that even though medication knowledge increased in the intervention group, adherence did not (Timmerman et al., 2016). A study utilizing the Medisafe app for three months participants (n = 50) indicated no improvement in medication knowledge. A limitation of this finding was that the study did not use a validated scale (Santo et al., 2019). Due to the heterogeneity of the samples, interventions, and measures of knowledge used, it is currently unclear if the passive provision of medication information through medication adherence smartphone apps in the form of written information or video influences patient’s perceived medication knowledge.

Tracking and reminders are a relatively common form of behavioral intervention within mobile health apps, including the Medisafe app. The use of trackers and reminders show generally small, positive associations with medication adherence (Peng et al., 2020). Two studies looked at the Medisafe app, both were focused on coronary artery disease/HTN and both found improvement in medication adherence but were not conducted in a medically underserved population and did not measure self-efficacy (Morawski et al., 2018; Santo et al., 2019). Another similar app was used across several chronic illnesses outside of the US. In contrast to the Medisafe app, this app had technical issues, with 50% of respondents saying they had problems with the reminders. Even so, medication adherence improved (Anglada-Martinez et al., 2016).

Although only a few studies have been conducted that specifically examine medication adherence apps with reminders with low-income medically underserved populations in the US, there have been promising results relating to medication adherence. However, none were found that examine reminders separately. Most of the studies found in this population focused on HTN, not multiple chronic illnesses. For example, Chandler et al. (2019) studied a culturally tailored app intervention for Hispanic individuals with uncontrolled HTN and found improvement in medication adherence. It incorporated tailored, culturally and patient relevant motivational messages, based on medication adherence scores from the previous day. The app interfaced with a blood pressure monitor and a medication tray which provided reminders in the form of blinking lights, chimes, and short message service (SMS) reminders and phone calls. There was significant involvement of HCPs and the conclusion was that the app was not scalable over multiple chronic illnesses (Chandler et al., 2019). A quality improvement project conducted in a rural health clinic in eastern Kentucky used Medisafe in a small group of patients (n = 14) with HTN and found that 50% improved both medication adherence and blood pressure classification by the 4–6-week follow-up visit (McManis et al., 2023). Another app, entitled Pill Phone, had similar attributes to the Medisafe app in terms of reminders, tracking and medication information and improved medication adherence in a high-risk urban population with HTN. This study provided phones for patients, which may have improved medication adherence as a result of being given a cell phone and contract at a time where cell phones were not as readily available as they are today (Patel et al., 2013). Some studies focused on specific medication regimens in diverse samples. One RCT measured adherence to oral pre-exposure prophylaxis therapy in a diverse cohort of men who have sex with men and used Artificial Intelligence (AI) automated directly observed therapy (DOT) which found no difference in medication adherence over six weeks (Buchbinder et al., 2023). In contrast, a small, prospective, single-arm study used an app enabled with an AI DOT platform as well as reminders proved more successful. 88% of participants enrolled in a methadone program in an urban setting in the US achieved greater than or equal to 80% medication adherence to Hepatitis C therapy using the app, which was defined as a high level of adherence (Litwin et al., 2020). A number of RCTs have been conducted in lower resourced international settings and have shown promising results in increasing medication adherence. An RCT conducted in Iran found the use of a mHealth app designed to manage HTN increased medication adherence through reminders, motivational messages, and access to educational content in patients seeking care in public hospitals (Bozorgi et al., 2021). Another RCT conducted in Vietnam implemented a mobile phone app which focused on HIV/AIDS treatment management in public and private hospital clinics in Hanoi and included a daily medication reminder that allowed healthcare personnel to monitor adherence. After one month of use, medication adherence increased significantly using a self-report scale (Tran et al., 2023). Additionally, an app with behavioral and educational components designed to improve medication adherence to epilepsy medications in a limited resource setting in India found a significant increase in medication adherence after three months using a self-report scale. The interactive medication reminder system was similar to the Medisafe reminder feature (Mirpuri et al., 2021). The current study adds important information regarding the use of medication adherence apps over a variety of chronic illnesses in a medically underserved population in the US, as opposed to only one specific chronic illness, which supports more widespread adoption.

Social support, particularly in the form of practical social support in chronic illness has been shown to be a positive influence on medication adherence (Dimatteo, 2004; Scheurer et al., 2012). Practical social support is defined as instrumental support, assistance, reminders from a social support person, organization, or support for a specific behavior (Dimatteo, 2004). A recent study among low socio-economic status tuberculosis patients in India found that instrumental support from family was associated with medication adherence (Nursasi et al., 2022). A systematic review concluded there are significant positive associations between social support of family and medication adherence in hypertensive patients, but no studies in the review included ethnic minority groups (Shahin et al., 2021). Yet in a cross-sectional study of young African American women with HTN, no association between social support and medication adherence was found (Spikes et al., 2020). This is in contrast to a texting intervention in non-Hispanic Black adults with uncontrolled HTN which found practical social support positively influenced medication adherence (Ferdinand et al., 2023). Furthermore, in a study using multicomponent apps in Black women with HTN, which included a medication adherence reminder on a Fitbit and social support, researchers found when the coaching intervention decreased and then stopped, use of the medication adherence feature decreased. The researchers attributed this to the benefits of providing social support through group coaching sessions which empowered participants through sharing experiences, problem-solving, committing to goals and being held accountable for progress, which may have encouraged app use (Abel et al., 2023). Levahot et al. (2011) found that medication specific social support, but not general social support, moderated the negative association between alcohol and other drug use and adherence to HIV medications (Lehavot et al., 2011). There is a need to study the effects of social support features within medication adherence apps (Etminani et al., 2020).

Commercially available medication adherence apps have not been widely studied in medically underserved populations, nor have they been studied across multiple disease states. To address these gaps, this RCT evaluated medication self-efficacy, medication knowledge, medication adherence and medication specific social support in medically underserved patients with chronic illness who used a mobile app which included access to medication information, reminders and tracking, and an optional social support feature.

## Methods

The study protocol was approved by the Vanderbilt University Institutional Review Board and is registered with clinicaltrials.gov (NCT05098743).

### Setting and participants

Participants were recruited from an FQHC. Recruitment took place from November 2021 through June 2022. Inclusion criteria were: (1) adults aged 18 years and older; (2) ability to speak and understand English; (3) personally own and use an Android (version 5.0 and up and at least 88 M of phone space) or Apple smartphone (iOS v13 or later and at least 165 MB of iPhone space) and (4) take at least 1 medication for a chronic illness. Patients were excluded if they: (1) were already using a medication reminder app or other electronic reminder system such as phone alarms; (2) owned a smartphone which was not capable of downloading the app; (3) had a diagnosis of severe dementia or serious mental illness, or (4) were otherwise unable to use a mobile phone or the medication reminder software either physically or cognitively.

Recruitment was conducted via an informational flyer, clinical referral form and in-person recruitment. The flyer/referral forms were available to clinicians, staff and patients in the FQHC clinician offices and at the reception desk. The form contained study information, the principal investigator (PI)’s contact information, and a place for patients interested in participation to provide their contact information. The form also allowed the referring HCPs signature verifying the patient’s medications listed in the EHR were correct and up to date. Patients who did not obtain their HCP signature were not able to participate in the research. The PI conducted in-person recruitment at the FQHC multiple days per week and worked closely with clinic staff to identify potentially eligible patients. The PI approached potentially eligible patients at the end of the health center (HC) visit.

### Data collection

All study data were collected and managed using REDCap electronic data capture tools hosted at Vanderbilt University. REDCap is a secure, web-based software platform designed to support data capture for research studies (Harris et al., 2009, 2019).

The PI confirmed participant eligibility before commencing with the study procedures. The PI discussed the study with and obtained informed consent from eligible patients who expressed interest with a signed referral form. Participants completed consent as either an IRB-approved e-consent form or a hard copy. All participants received a copy of the consent form.

Following consent, participants completed the baseline survey on an iPad. All patients received a copy of their medication list from the EHR from their HCP or HC staff and reviewed the confirmed medication list with study personnel. If discrepancies were identified between perceived current medications and the EHR-generated list, the research staff resolved the discrepancy with the HCP and participant. Following baseline procedures, participants were randomized to either a control or an app intervention group by REDCap. The intervention group used the medication adherence app for one month and the control group received a printed out copy of their medication list that the PI reviewed with them. The one month time frame was chosen as previous app studies have shown significant change in this timeframe (Svendsen et al., 2018). Also, Morawski et al. (2018) measured adherence and blood pressures while using the Medisafe app at 4, 8 and 12 weeks and blood pressures decreased and then leveled out at 4 weeks, indicating initial effects are seen within this period (Morawski et al., 2018). All participants were contacted by phone or text within the first week of enrolling in the study to address potential questions regarding the app (intervention group) or their medications (control group).

To reduce the likelihood of missing data, participants were provided three options for completion of the one-month follow-up survey: a web-based survey, by phone, or in-person at the HC. Those who preferred the web-based survey received the survey link via REDCap. Those who preferred a telephone call received a hard copy of the follow-up survey during the initial visit. During the telephone call the questions were read to participants and their answers were recorded in the REDCap database. Participants who did not complete the follow-up survey within 10 days of the one-month follow-up date, received two reminders via phone, email, or text messaging based on their communication preference. Participants received a $25.00 gift card following completion of the baseline survey and a $35.00 gift card after completion of the follow-up survey.

### Medisafe app

The Medisafe app is a Health Insurance Portability and Accountability Act (HIPAA) compliant medication adherence app that is commercially available at no cost in the iTunes and Google app stores. The language mode of the app can be switched, if desired. The Medisafe app provides interactive and customizable daily, timed reminders to reinforce medication taking at a set time every day through a push notification, equivalent to an alarm or text message. These reminders can be snoozed, rescheduled and/or marked as taken or skipped. Scheduled reminders occur three times every ten minutes. Additional functionality features include the ability to snooze or delay the reminder for a preset amount of time, and a medication database which includes written and video content. The app also incorporates features that support retrospective memory such as being able to track medications based on responses to the reminders, view adherence statistics, share data and offer multiple user support (Parra-Calderón et al., 2016). Refill reminders are set by entering the number of pills left. If using the Medfriend feature, the app will alert the designated Medfriend through a push notification on their phone, who can provide peer support and additional reminders through text messages, email or a telephone call if the patient misses a dose. Medfriends also have access to the medication list of the patient.

### Study conditions

#### App intervention

Research staff assisted the patient with downloading and setting up the Medisafe app on their smartphone at the HC. Participants were shown how to use the app using written educational materials. Participants entered their medication list into the app and were shown how to choose their desired language for using the app. Medications entered into the app were confirmed against the printed medication list from the EHR using read-back technique. Participants were shown (1) how to enter the medication name, dosage, medication appearance (color and format of pill), frequency (every day, every other day, specific days of the week, etc.), the time the medication was regularly taken, and the number of pills left for the refill reminder (if the patient wanted this option) (2) how to access their medications in the app, how to access information (e.g., side effects, medication interactions) about the specific medication, how to edit a medication, if necessary, and how to indicate when a medication was taken, skipped or rescheduled when they received a reminder alarm to take their medication (3) the adherence reports and the Medfriend feature. (4) Finally, participants were shown how to access the help and support section. After completing the follow-up questionnaire, participants were reminded they were welcome to continue using the app if they wanted to, but the research study was completed.

#### Control

The control group participants received a printed copy of their medication list from the EHR from their HCP or HC staff and were guided through the same medication review procedures as the app intervention group by research personnel. At follow-up, participants in the control group were asked for feedback on the usefulness of the printed medication list and to describe how or if they used it to facilitate medication management. After completing the follow-up questionnaire, participants were informed about their option to download the mobile app. If participants were interested, they were assisted with setting up the app, as described above.

### Measures

The primary outcomes for this study were medication adherence, medication self-efficacy, medication knowledge and medication social support.

#### Medication adherence

The Adherence to Refills and Medications Scale (ARMS) assesses both medication and refill adherence. It is a 12-item scale with total scores ranging from 12 to 48 and has been validated for low-literacy chronic disease populations. Lower scores are indicative of better adherence. Internal reliability of the scores in this study were consistent with previously reported reliabilities (Cronbach’s alpha: baseline = 0.88, follow-up = 0.74) (Kripalani et al., 2009). Two participants did not complete one of the 12 items in this measure. Their 11-item total score was prorated to the scale of total scores based on 12-items.

#### Medication self-efficacy

The Self-efficacy for Appropriate Medication Use Scale (SEAMS) was used to measure self-efficacy for medication adherence. The measure has been validated and is reliable in low-literacy chronic disease populations (Risser et al., 2007). Responses to the 13 items are summed to generate possible total scores ranging from 13 to 39. Higher scores indicate higher levels of self-efficacy for medication adherence. Reliabities of the scores generated in this study at baseline and follow-up were both 0.92 (Cronbach’s alpha). One participant did not complete one of the 13 items in this measure. Their 12-item total score was prorated to the scale of total scores based on 13-items.

#### Medication knowledge

Patient-perceived medication knowledge was assessed using the Okere-Renier Survey (Okere et al., 2014). The knowledge scale is comprised of five, 5-point Likert scales with response options of “strongly disagree” to “strongly agree.” The questions relate to general knowledge (how and when to take medications) and understanding of drug interactions and side effects (Okere et al., 2014). Scores in this study had a reliability of 0.81 at baseline and 0.77 at follow-up, which are consistent with previously reported reliabilities (Okere et al., 2014).

#### Medication Social Support

Medication specific social support was assessed using a measure originally developed to identify perceptions of social support in persons living with HIV (Lehavot et al., 2011). Response options for the 8-items range from 0 (never) to 4 (very often). Items were averaged to form mean total score ranging from 0 to 4, with higher scores reflecting more social support. In this study, the reliability of scores were Cronbach alphas of 0.87 (baseline) and 0.92 (follow-up), both consistent with previously reported reliabilities (Lehavot et al., 2011).

### Data analysis

An intent-to-treat analysis was conducted using IBM SPSS Statistics (v. 28). Based on a pilot study for this research we anticipated that Cohen’s *d* effect sizes would range from 0.41 (ARMS) to 0.69 (SEAMS) (Hartch, 2022, March 31 - April 1). Assuming similar distributions of baseline scores to those observed in the pilot study and a similar correlation of ~ 0.30 between the baseline and follow-up scores, a total sample of 60 participants was estimated to enable detection of differences between the groups as small as Cohen’s *d* values of 0.6–0.7 (80% power, alpha = 0.05).

Frequency distributions were used to summarize nominal/ordinal data. Due to skewed continuous data distributions, median (IQR) were used for those summaries. Study group differences in characteristics at baseline were assessed using Chi-Square (nominal/ordinal data) and Mann-Whitney (continuous) tests. Effects of the Medisafe app on the study outcome measures were tested using multiple linear regression models. Each model included an outcome measure as the dependent variable and the study group as the critical independent variable. To control for individual differences in baseline values, those models included the respective outcome variable baseline value as a covariate. Skewed distributions were transformed to normal prior to conducting regression analyses. The adjusted study group beta-coefficients were transformed to Cohen’s *d* effect statistics. The criteria for statistical significance used a maximum alpha of 0.05 (p < .05).

Given measurement error inherent in self-report measures and the desire to assess change beyond what could be due to simple measurement error, a reliable change index (RCI) criterion was generated for each outcome (Christensen & Mendoza, 1986; Jacobson et al., 1984). A separate 95% confidence change criterion value was generated for each outcome measure using the respective scores’ reliability coefficient (Cronbach’s alpha) and the variability of the scores at baseline (see referenced work for specific formula). Each patient’s raw change value was evaluated against that criterion to determine whether the amount of change was greater than the criterion value for reliable change in that measure’s scores and the direction of that change. The evaluations resulted in three categories of scores: (1) those that decreased between the times of assessment more than could be expected given baseline variability and reliability of the scores with 95% confidence, (2) those that increased between the times of assessment more than could be expected given baseline variability and reliability of the scores with 95% confidence, and (3) those that did not change beyond what would be expected given baseline variability and reliability of the scores. Chi-square tests of independence were used to test for differences in proportions of reliable change in the hypothesized direction between the two groups.

## Results

### Participant characteristics

**Fig. 1:**
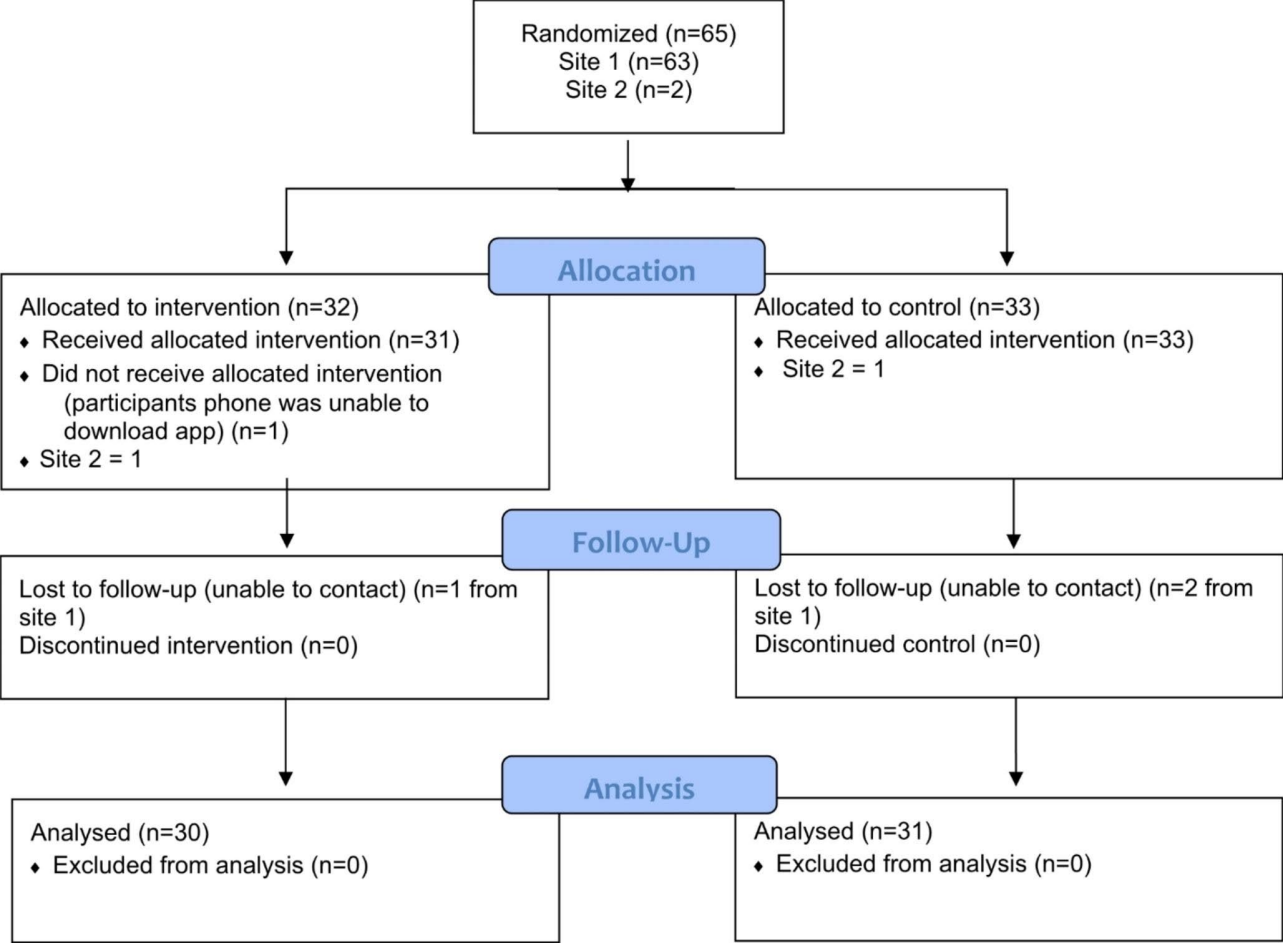
Flowchart of Study Participants

Originally, 65 consented participants were randomized (32 app, 33 control). One participant had no data because they were unable to download the app despite multiple attempts and one participant was lost to follow-up. Two participants were lost to follow-up in the control group. Thus, the analysis sample was comprised of 30 participants in the intervention (app) group and 31 in the control. A CONSORT flow diagram is shown in Fig. 1.

Table 1 summarizes the characteristics of the participants. The characteristics were very similar for both groups with no differences being statistically significant (p > .09). The majority of the sample was non-White (84.7%), with government insurance (75.4%) while 11.5% were uninsured. Of those who responded (*n =* 24), 50% made less than $25,000/year. The median Brief Health Literacy Summary score for the participants was 12.0 [IQR = 7.5, 15.0] with a range of 3 and 15, which is generally high and did not differ between groups (p = .514). The most common chronic illnesses were HTN (70.5%) followed by hyperlipidemia (63.9%) and D2M (49.2%).

**Table 1:** Sociodemographic Characteristics at Baseline

Characteristic	Overall n (%) (N = 61)	Control n (%) (N = 31)	Intervention n (%) (N = 30)	*p*-value
**Race/Ethnicity**	N = 59	N = 30	N = 29	0.266
Asian	7 (11.9)	2 (6.7)	5 (17.2)	
Black or African American	23 (39.0)	13 (43.3)	10 (34.5)	
Hispanic/Latino	14 (23.7)	10 (33.3)	4 (13.8)	
Native American or Alaska Native	1 (1.7)	0 (0)	1 (3.4)	
White	9 (15.3)	3 (10.0)	6 (20.7)	
Other^a^	5 (8.5)	2 (6.7)	3 (10.3)	
**Marital Status**				0.193
Married/Partnered	23 (37.7)	8 (25.8)	15 (50.0)	
Separated or Divorced	5 (8.2)	4 (12.9)	1 (3.3)	
Widowed	2 (3.3)	1 (3.2)	1 (3.3)	
Single/Never married	31 (50.8)	18 (58.1)	13 (43.3)	
**Employment Status**	N = 61	N = 31	N = 30	0.243
Employed	24 (39.3)	9 (29.0)	15 (50.0)	
Unemployed	30 (49.2)	18 (58.1)	12 (40.0)	
Retired	7 (11.5)	4 (12.9)	3 (10.0)	
**Education**				0.492
Some high school or less	14 (23.0)	6 (19.4)	8 (26.7)	
High school graduate	15 (24.6)	11 (35.5)	4 (13.3)	
College credit, no degree	15 (24.6)	7 (22.6)	8 (26.7)	
Trade/vocational training	7 (11.5)	3 (9.7)	4 (13.3)	
Associate’s degree	4 (6.6)	2 (6.5)	2 (6.7)	
Bachelor’s degree or more	6 (9.8)	2 (6.5)	4 (13.3)	
**Difficulty Paying Bills**	N = 57	N = 28	N = 29	0.090
Very difficult	9 (15.8)	3 (10.7)	6 (20.7)	
Somewhat difficult	27 (47.4)	17 (60.7)	10 (34.5)	
Not very difficult	10 (17.5)	2 (7.1)	8 (27.6)	
Not at all difficult	11 (19.3)	6 (21.4)	5 (17.2)	
**Type of Health Insurance**	N = 61	N = 31	N = 30	0.347
Uninsured (Sliding Scale)	7 (11.5)	5 (16.1)	2 (6.7)	
Government Insurance	46 (75.4)	21 (67.7)	25 (83.3)	
Private Insurance	8 (13.1)	5 (16.1)	3 (10.0)	
**Current Chronic Illnesses**	N = 61	N = 31	N = 30	
Hypertension	43 (70.5)	21 (67.7)	22 (73.3)	0.632
Diabetes Type 2	30 (49.2)	16 (51.6)	14 (46.7)	0.699
Hyperlipidemia	39 (63.9)	20 (64.5)	19 (63.3)	0.923
Asthma	8 (13.1)	3 (9.7)	5 (16.7)	0.419
Other^b^	20 (32.8)	9 (29.0)	11 (36.7)	0.525

Italian, Iberian and Scottish (1), Black and Native American, Haitian (1), Jamaican (1), Puerto Rican, Black, White (1) b. Anxiety (2), Arthritis (chronic pain) (1), Blood clotting disorder (2), Cirrhosis (1), Depression (2), COPD (2), Diabetes Type 1 (1), GERD (1), Gout (1), Heart Disease (2), Hypothyroidism (3), Fibromyalgia (1), Idiopathic Intracranial Hypertension (1), Irritable Bowel Syndrome (1), Lupus (1), Rheumatoid Arthritis (1), Thyroid Disorder (1).

Summaries of the study outcome measures scores at baseline are summarized in Table 2. While there was considerable variability in all of the measures scores, values for the ARMS (medication adherence) were generally low, indicating higher levels of medication adherence and the SEAMS scores tended to be in the higher levels of medication self-efficacy. Medication knowledge also tended to be in the higher levels of knowledge, yet medication social support was very low. Scores for all of the measures for both groups were quite similar and no statistically significant differences at baseline were observed (p > .30).

**Table 2:** Summaries of Study Outcome Measures by Time of Assessment for Control (N = 31) and Intervention (N = 30) Study Groups

Measure	Baseline^a^ Median [IQR]	Post Median [IQR]	Change Median [IQR]	*p*-value^b^	Effect Size^c^
Medication adherence ARMS				0.014	-0.52
Control	17.0 [14, 21]	15.0 [13, 18]	-1.0 [-3, 0]		
Intervention	18.5 [14, 25]	14.0 [12, 17]	-4.0 [-8, -1]		
Medication self-efficacy SEAMS				0.035	0.43
Control	31.0 [26, 39]	33.0 [26, 39]	0.0 [-3, 3]		
Intervention	29.0 [25, 36]	35.5 [30, 39]	3.5 [0, 8]		
Medication knowledge				0.863	0.20
Control	21.0 [16, 24]	22.0 [19, 25]	0.0 [-3, 4]		
Intervention	20.5 [14, 24]	23.5 [17, 25]	2.0 [0, 56]		
Medication social support				0.560	0.11
Control	1.3 [0.3, 2.4]	0.6 [0.2, -1.8]	-0.1 [-0.5, 0.2]		
Intervention	1.1 [0.5, 2.0]	0.6 [0.0, 2.0]	0.0 [-0.7, 0.5]		

^**a**^ No statistically significant differences between groups (p > .30)

^b^Test of group differences at post controlling for pre-test scores

^c^Cohen’s d statistic generated from regression coefficient

### Primary outcomes

#### Medication adherence

As shown in Table 2, compared to the wait list control group, the app group had a significantly greater decrease in the ARMS (median = -4.0 vs. -1.0, *p* = .014, Cohen’s *d* = -0.52), indicating greater medication adherence. Given the reliability of the medication adherence scores and the variability of the scores at baseline, a reliable change criteria for the scores in this study was ± 5.98. Almost half of patients in the intervention group (43.3%, n = 13) exceeded that criteria for demonstrating a reliable increase in adherence compared to only 9.7% (n = 3) in the control group (*p* = .003).

#### Medication self-efficacy

In general, there was no change in SEAMS scores for the control group (median = 0.0, IQR= -3, 3), while participants given the app had a median increase of 3.5 points (IQR = 0, 8, *p* = .035, Cohen’s *d* = 0.43). Given the reliability of the SEAMS scores and the variability of the scores at baseline, RCI criteria for those scores in this study was ± 5.45. As noted above, self-efficacy for taking medications was generally high for both groups at baseline yet 36.7% (n = 11) of participants in the intervention group exceeded the criteria for reliable change in the direction of increasing self-efficacy compared to only 12.9% (n = 4) in the control group (*p* = .065).

#### Medication knowledge

40% (n = 12) of the participants stated that they accessed educational information, however data from the Medisafe app did not confirm this. Those records indicated that three participants accessed the supplemental education information on a total of fourteen different medications. As shown in Table 2, no significant effect of the app on knowledge was observed in this study (*p* = .863, Cohen’s *d* = 0.20). The RCI criteria for the medication knowledge scores in this study was ± 6.10. Approximately 13% (n = 4) of participants in the intervention group exceeded that criteria for an increase in knowledge compared to 16.1% (n = 5) in the control group (*p* = .337).

#### Medication Social Support

Usage of the Medfriend option was low. Medisafe data confirmed that only one participant set up this feature. The other participant who reported using the feature stated her husband set up the app and designated her as a Medfriend, which explains why Medisafe did not report this second user. No statistically significant effect of the app was observed on medication social support (*p* = .560, Cohen’s *d* = 0.11). The RCI criteria for the medication social support scores in this study was ± 1.01. Approximately 13.3% (n = 4) of participants in the intervention group exceeded that criterion for an increase in medication social support compared to 3.2% (n = 1) in the control group (*p* = .347).

## Discussion

We hypothesized participants randomized to the mobile phone app condition would report increased medication adherence and medication self-efficacy compared to participants in the control condition after one month. Our results supported those hypotheses. We also hypothesized that participants randomized to the mobile phone app condition would report increased patient-perceived medication knowledge and medication social support compared to participants in the control condition. Our results did not demonstrate those hypothesized effects. The results did indicate positive impact in several areas including that the Medisafe app was associated with improved self-reported medication adherence and medication self-efficacy and is feasible for delivery in a diverse sample of patients with chronic illness in an FQHC setting. Additionally, there was a very low rate of attrition, strengthening the case for implementation of this intervention.

Regarding medication adherence, those using the Medisafe app demonstrated significant improvement compared to the controls. A higher percentage of the participants using the app demonstrated an increase in medication adherence above and beyond what could be expected given measurement error compared to those in the control group (43% vs. 10%). No group differences were found in medication knowledge nor medication social support, despite the integration of app features designed to support those outcomes. To our knowledge, this is the first study to test the impact of specific app features on medication adherence, self-efficacy, knowledge, and social support separately in a medically underserved population across a range of chronic illnesses.

All app users received audible and visual reminders tailored to their medications and schedules which may have had a significant positive association with medication adherence. Apps that include reminders have been found to increase medication adherence particularly in patients with lower adherence scores at baseline (Moorhead et al., 2017; Morawski et al., 2018). Some researchers postulate that adherence effect sizes may have been influenced by a ceiling effect in samples of more adherent patients (Anglada-Martinez et al., 2016; Santo et al., 2019). Comparison with prior studies is constrained, as none have studied the medically underserved with chronic illnesses. Inclusion of a variety of chronic illnesses limits generalizability to specific diseases but provides greater generalizability to research on chronic illnesses as a whole.

Much research based on Social Cognitive Theory (SCT) has identified self-efficacy as important for understanding adherence (Bandura, 2004; Flower et al., 2016; Huang et al., 2018; Lee et al., 2013; Meinema et al., 2015). However, little evidence exists to replicate those findings using mobile apps or technologies. That dearth of evidence points to self-efficacy as potentially a valuable consideration in app design and improvement of adherence in mHealth research. Notably, the small (n = 10) pilot study for this RCT also indicated an increase in self-efficacy with a 2-week duration of app use (*d* = 0.69) (Hartch, 2022, March 31 - April 1). Our findings contrast to those observed from an D2M app study which found no effect on medication self-efficacy (Kjos et al., 2019). Although SEAMS scores in this study were generally high, the intervention group’s increase in medication self-efficacy and adherence is supported by the conceptual framework using SCT. The framework posits that self-efficacy is influenced by individual factors as well as barriers and facilitators in the environment (LaMorte, 2019). Our finding is particularly salient for the medically underserved population who, in general, have many barriers to medication adherence related to SDOH. It is possible that by adding an audible and visual reminder into the participant’s daily living environment, increased feelings of self-efficacy were facilitated.

Use of the app did not have a significant impact on participant’s self-reported medication knowledge. This could possibly be due to ceiling effects, as scores were generally high. The knowledge that participants could have gained by using the app included how and when to use their medications, food and beverage interactions with their medications, as well as side effects of medications. According to Medisafe data, few participants accessed the medication information card and/or videos available to them over the study period, but participants self-reported accessing these features at a greater rate. Some cross-sectional correlational studies have found higher levels of medication knowledge associated with higher levels of medication adherence in diverse samples of patients with a variety of chronic illnesses (Fernandez-Lazaro et al., 2019; Najjar et al., 2015). No studies were found that showed app use positively influenced medication knowledge. Our study supports prior research suggesting that knowledge alone does not improve adherence, and that mechanisms other than knowledge influence adherence.

Medication social support was not influenced by Medisafe app use. Feelings of medication social support, which were low at baseline, showed no improvement. Our study was the first of its kind to assess the impact of a social support feature operationalized in the app. An important consideration was that Medisafe data showed that only one participant downloaded the social support feature, although one participant stated her husband downloaded the app and made her his Medfriend. The very low usage of Medfriend feature prevented an assessment of an impact of that feature, app usage alone did not increase feelings of social support.

The lack of uptake of the social support feature may have resulted from participants not wanting to burden family and friends with reminders, or not wanting to share their medication list with others. Patients with chronic illness have expressed not wanting to bore or annoy people in their surroundings but rather appreciated sharing experiences in online communities (Kraan et al., 2008). Research indicates that online peer-to-peer communities provide supportive space for daily self-care in chronic illness and strengthen social ties and alleviate strains on relationships with family and friends (Kingod et al., 2017). Additional research supports that relationships with family and friends were actually strengthened for those who participated in online social support groups (Chung, 2013). Modifications to the Medfriend feature, which currently targets family and friends, may need to be made to increase uptake of this feature.

### Future directions

Future research on medication social support could be further enhanced to include caregivers. For example, implementing the app in adolescents who designate a parent as a Medfriend to ascertain if there is greater uptake of the feature and if it influences medication adherence is an avenue to explore. Additionally, caregiver burden in adults who serve as caregivers for other adult or elderly patients with chronic illness could be measured in those using the Medfriend feature. Formative research is needed to understand how to design mobile technologies to improve practical social support and improve user uptake for populations who would benefit from increased social support (Konstantinou et al., 2020). There may be other methods for operationalizing social support into a feature that will be more palatable to a chronically ill population. Instead of designating specific Medfriends, which were identified as family members in this study, an online support group of app users could be designed to facilitate greater usage of a similar feature. Instead of the social support person having access to the person’s medication list, a more general adherence text reminder when the patient doesn’t take their medications could alleviate possible confidentiality concerns. It is possible that fewer or more general messages in the context of inadequate adherence would be more salient and acceptable. A longitudinal prospective study of medication adherence to cardiovascular medications in participants with T2D in an underserved urban community found participants identified the need for technological innovation, including apps, as well as community linkages as an important means of social support to share ideas and provide accountability for medication adherence (McClintock et al., 2022). This finding and results of this research support the recommendation that more research is needed to discover how to design and/or implement a social support feature in a way that encourages user engagement and fosters social support.

The implications of these findings point towards a possible large-scale benefit of using this commercially available, free medication adherence app in medically underserved populations. To improve dissemination, future research and implementation studies could incorporate the use of a provider portal. Such a portal could provide adherence reports to HCPs that would serve as another tool to enhance dialogue and encourage medication adherence during telehealth and office visits. Comparing participants who use the app independently, as they did in this study, with patients whose providers use the provider portal and measuring outcomes would shed new light on the phenomenon. The experiences of providers using the portal would be important to capture for feasibility of the intervention. Two barriers mentioned when implementing texting interventions in FQHCs included the inability of some of the sites to integrate the texting platforms with EHRs, which made tracking and documenting texts labor-intensive, and raised concerns regarding HIPAA violations (Garg et al., 2016). Additionally, a qualitative study of practitioners’ perspectives on an mHealth digital health intervention to support medication adherence and blood pressure management in Ireland found concerns regarding increased workload associated with managing information and increased healthcare use by the “worried well” (Morrissey et al., 2018). Newer EHR technologies provide integrated SMS features. These have yet to be fully utilized for health behavior tailoring or change but may provide the most feasible option for using SMS to support medication adherence in routine care. Patients and providers have expressed enthusiasm for SMS in routine care and that approach is aligned with patient-centered care (Garg et al., 2016).

Challenges for widespread implementation of the intervention in a busy FQHC setting include personnel and funding. Onboarding patients to demonstrate proper app use and using hands-on exercises, as well as prescribing an app have all been suggested to increase patient success (Chan et al., 2017). Keeping staff informed and trained to participate in app interventions can be challenging due to high staff turnover (Olomu et al., 2022). It has been suggested that implementation and monitoring of apps in FQHC primary care settings should be assigned to individuals other than physicians or nurse practitioners (Mares et al., 2016). Nurses and assistive personnel such as medical assistants may be better able to incorporate implementation of apps into their workflow, and partnerships with universities or local health departments have been suggested to provide students or personnel to assist in implementation (Avegno et al., 2023). Other challenges include maintenance of the app over time, making partnerships with the developer and contracts to provide long-term support needed. Finally, cost effectiveness of apps needs to be demonstrated in order to obtain third party reimbursement for these services, which highlights this as an area of future policy research.

### Limitations

Although our study addressed several gaps in the mHealth research base, we acknowledge several limitations. The study included a small sample and participants were only followed for one month. Due to the small sample size, we cannot determine the specific role of self-efficacy as a mediator. The need for studies of longer duration to measure outcomes and engagement are necessary. Also, self-reported measures may be subject to social desirability bias and may overestimate adherence. Insights regarding the relation of social support to medication adherence were not possible due to lack of uptake of the feature, but this is a finding in and of itself. A strength of this study was that participants were able to use the app for multiple types of medications and chronic illnesses. However, because of this, clinical outcomes were not measured across the various disease states.

## Conclusions

Our study found that a medication adherence mobile app can improve medication adherence in a medically underserved population with chronic illness seeking care in an FQHC. The mechanism of impact may be mediated by self-efficacy (Bandura, 2004). As there was limited uptake of the social support feature, new ways to encourage social support within medication adherence apps may facilitate medication adherence further. As smartphone ownership and use continues to rise in this population, this free and simple technology can be implemented widely in the FQHC clinical setting to assist patients in adhering to their medication regimen. As community health centers care for over 30 million patients annually, (National Association of Community Health Centers, 2022), the impact of using this medication adherence mobile app could have far reaching positive effects on the health of medically underserved patients with chronic illness in the US.

## Data Availability

Limited de-identified data is available upon request from the corresponding author.

## Declarations

None to declare.
